# A163 EVALUATING THE ASSOCIATION BETWEEN PERIPHERAL BLOOD EOSINOPHILS AND DRUG RESPONSE IN CROHN’S DISEASE: A PRELIMINARY REPORT

**DOI:** 10.1093/jcag/gwab049.162

**Published:** 2022-02-21

**Authors:** B Cai, A Wilson

**Affiliations:** Gastroenterology, Western University, London, ON, Canada

## Abstract

**Background:**

Th1, Th2, and Th17 immune pathways are variably activated in inflammatory bowel disease (IBD). The degree to which pathway having a more dominant role in propagating Crohn’s disease (CD) is not considered when selecting a treatment strategy. Th2 cytokines, IL-5 and IL-13 enhance eosinophil survival, recruitment and degranulation, facilitating inflammation. Mucosal eosinophilia has been documented in CD and its presence is a surrogate marker of Th2 pathway activation. Peripheral eosinophilia has an established role in asthma to help prognosticate treatment response to Th2-cytokine-specific therapies. We hypothesize the pattern of peripheral blood eosinophils (PBE) at CD diagnosis will identify distinct subsets within a larger CD population and correlate with response to treatments such as prednisone or anti-TNFs.

**Aims:**

We aim to evaluate the pattern of PBE of CD patients at time of diagnosis (prior to drug exposure) and with each subsequent treatment; and if baseline PBE or any changes seen with drug exposures are predictive of treatment response.

**Methods:**

A retrospective cohort study is ongoing with CD patients exposed to glucocorticoids and an anti-TNF seen at one of 3 hospitals affiliated with University of Western Ontario. Patients were identified using administrative databases and reviewed for biochemical data (complete blood count) and disease activity (Harvey Bradshaw Index) at baseline as well as before and after each drug exposure. Participants were classified as having high PBE (eosinophils >200 cells/μl) versus low PBE (eosinophils <200 cells/μl).

**Results:**

To date,10 of 200 CD patients are included in the preliminary analyses with a mean age of 47. 8 had PBE *>*200 cells/μL at baseline, while 2 did not. The median number therapies used was 4 (IQR=0.75). All received glucocorticoids followed by an anti-TNF. There was no difference in the occurrence of hospitalization or surgery between the two cohorts. Overall 75% (n=6/8) participants with high PBE had clinical response to glucocorticoid exposure, seen as a 3-point decrease in HBI compared to 0% (n=0/2, p=0.5) in the low PBE cohort. With subsequent anti-TNF exposure, PBE rebounded in 6 participants. More patients in the high PBE group required anti-TNF dose escalation versus the low PBE group (63%, n=5/8 versus 50%, n=1/2, p=0.99). The proportion of patients with anti-TNF discontinuation was similar in both groups (high PBE, 50%, n=4/8 versus low PBE, 50%, n=1/2, p=1.00).

**Conclusions:**

Peripheral eosinophilia is seen in varying degrees in CD patients. Participants with high PBE appear to be more steroid-responsive which is typical for Th2-mediated pathways. They were less responsive to Th1-targeting anti-TNF therapies, requiring more dose-escalation and discontinued anti-TNF treatment. Completion of this study will help clarify the association between PBE in CD and treatment response.

**Funding Agencies:**

None

